# Effect of JAK inhibitors on the three forms of bone damage in autoimmune arthritis: joint erosion, periarticular osteopenia, and systemic bone loss

**DOI:** 10.1186/s41232-023-00293-3

**Published:** 2023-09-19

**Authors:** Masatsugu Komagamine, Noriko Komatsu, Rui Ling, Kazuo Okamoto, Shi Tianshu, Kotaro Matsuda, Tsutomu Takeuchi, Yuko Kaneko, Hiroshi Takayanagi

**Affiliations:** 1 Department of Immunology, Graduate School of Medicine and Faculty of Medicine, The University of Tokyo, Tokyo, Japan; 2 Division of Rheumatology, Department of Internal Medicine, Keio University School of Medicine, Tokyo, Japan; 3 Department of Osteoimmunology, Graduate School of Medicine and Faculty of Medicine, The University of Tokyo, Tokyo, Japan; 4 Saitama Medical University, Saitama, Japan

**Keywords:** JAK inhibitor, Autoimmune arthritis, Bone damage, Osteoclast, Osteoblast

## Abstract

**Background:**

The types of bone damage in rheumatoid arthritis (RA) include joint erosion, periarticular osteoporosis, and systemic osteoporosis. Janus kinase (JAK) inhibitors ameliorate inflammation and joint erosion in RA, but their effect on the three types of bone loss have not been reportedly explored in depth. We aimed to clarify how JAK inhibitors influence the various types of bone loss in arthritis by modulating osteoclastic bone resorption and/or osteoblastic bone formation.

**Methods:**

Collagen-induced arthritis (CIA) mice were treated with a JAK inhibitor after the onset of arthritis. Micro-computed tomography (μCT) and histological analyses (bone morphometric analyses) on the erosive calcaneocuboid joint, periarticular bone (distal femur or proximal tibia), and vertebrae were performed. The effect of four different JAK inhibitors on osteoclastogenesis under various conditions was examined in vitro.

**Results:**

The JAK inhibitor ameliorated joint erosion, periarticular osteopenia and systemic bone loss. It reduced the osteoclast number in all the three types of bone damage. The JAK inhibitor enhanced osteoblastic bone formation in the calcaneus distal to inflammatory synovium in the calcaneocuboid joints, periarticular region of the tibia and vertebrae, but not the inflamed calcaneocuboid joint. All the JAK inhibitors suppressed osteoclastogenesis in vitro to a similar extent in the presence of osteoblastic cells. Most of the JAK inhibitors abrogated the suppressive effect of Th1 cells on osteoclastogenesis by inhibiting IFN-γ signaling in osteoclast precursor cells, while a JAK inhibitor did not affect this effect due to less ability to inhibit IFN-γ signaling.

**Conclusions:**

The JAK inhibitor suppressed joint erosion mainly by inhibiting osteoclastogenesis, while it ameliorated periarticular osteopenia and systemic bone loss by both inhibiting osteoclastogenesis and promoting osteoblastogenesis. These results indicate that the effect of JAK inhibitors on osteoclastogenesis and osteoblastogenesis depends on the bone damage type and the affected bone area. In vitro studies suggest that while JAK inhibitors inhibit osteoclastic bone resorption, their effects on osteoclastogenesis in inflammatory environments vary depending on the cytokine milieu, JAK selectivity and cytokine signaling specificity. The findings reported here should contribute to the strategic use of antirheumatic drugs against structural damages in RA.

**Supplementary Information:**

The online version contains supplementary material available at 10.1186/s41232-023-00293-3.

## Background

Rheumatoid arthritis (RA) is an autoimmune disease characterized by inflammation and bone damage. Three forms of bone damage, joint erosion, periarticular osteoporosis and systemic osteoporosis are observed in RA [1–3]. In inflamed synovium, activated immune cells, including T cells, B cells and macrophages, produce pro-inflammatory cytokines which stimulate synovial fibroblasts to express receptor activator of NF-κB ligand (RANKL), which is essential for osteoclast formation, leading to a progression of joint erosion [4–8]. Periarticular osteoporosis is observed in the trabecular bone proximal to the inflamed joints [9, 10]. Although the mechanism of periarticular osteoporosis has not been fully elucidated, it is reported that plasma cells contribute to periarticular osteopenia by expressing RANKL, autoantibodies, and pro-inflammatory cytokines [11, 12]. Systemic osteoporosis, which is often observed in the vertebrae, can be a risk factor for bone fracture in RA patients [13, 14]. Aging, menopause, and vitamin D deficiency cause osteoporosis in general. In addition, systemic osteoporosis in RA is promoted by activation of the immune system, glucocorticoid treatment, and immobility [3, 15]. Biologic disease modifying anti-rheumatic drugs (bDMARDs), such as TNF inhibitors and IL-6 inhibitors, are widely used and effectively ameliorate joint inflammation and erosion [15]. JAK inhibitors are representative of targeted synthetic DMARDs (tsDMARDs) which suppress joint inflammation and erosion to an extent similar to bDMARDs [16–18]. JAKs phosphorylate signal transducer and activator of transcription (STAT) proteins, which then translocate to the nucleus and regulate the transcription of genes involved in various cellular responses. JAKs (including JAK1, JAK2, JAK3, and TYK2) are ubiquitously expressed in various types of cells, including immune cells, synovial cells, osteoclasts, and osteoblasts, which together constitute the triangular immune-fibroblast-bone interaction underlying the bone damage in RA [19–21]. Thus, it is important to elucidate the effect of JAK inhibitors on bone damage, by paying attention to shedding light on their effects on the different forms of arthritis-related bone loss.

The in vitro effects of JAK inhibitors on immune cells and synovial fibroblasts have been intensively studied. It has been shown that JAK inhibitors suppress cytokine production and the activation of synovial fibroblasts as well as immune cells such as CD4^+^ T cells and DCs [22–24]. Compared with the wealth of literature on the effect of JAK inhibitors on immune cells and synovial fibroblasts, there are few reports on the effect of JAK inhibitors on bone cells [18–25].

Bone homeostasis is maintained by the balance between the bone resorption by osteoclasts and bone formation by osteoblasts [3]. Osteoclasts are unique, multinucleated bone-resorbing cells which differentiate from bone marrow-derived monocyte/macrophage lineage cells. Osteoblasts, which are of mesenchymal origin, form bone by producing bone matrix proteins and mediating mineralization. Osteocytes embedded in the bone matrix as well as osteoblastic cells express RANKL and stimulate the osteoclastogenesis necessary for bone renewal under physiological conditions [26, 27]. Under inflammatory conditions, osteoclast formation is promoted, whereas osteoblastic bone formation is impaired, tipping the balance in favor of resorption [28, 29]. Thus, it is important to clarify the in vivo effect of JAK inhibitors on both osteoclasts and osteoblasts when investigating on the effect of JAK inhibitors on the various types of bone damage in RA.

It is reported that JAK inhibitors inhibit osteoclastogenesis by suppressing RANKL expression on osteoblastic cells, but it has also been shown that JAK inhibitors do not affect osteoclastogenesis when added to the monoculture of osteoclast precursor cells [30, 31]. In contrast, a recent study reported that JAK inhibitors inhibit migration of osteoclast precursors and function of osteoclasts under LPS-induced inflammatory conditions in vivo [32]. In addition, JAK inhibitors promote osteoblastogenesis in part by increasing the expression of bone anabolic proteins in osteoblasts in vitro and there is a report in which JAK inhibitors reversed bone erosion in cases of RA [30]. Administration of JAK inhibitors into mice at the induction of arthritis suppressed bone erosion and periarticular osteopenia, while administration of a JAK inhibitor after the onset of arthritis showed less effect on bone erosion [30, 33]. However, the in vivo effect of JAK inhibitors on osteoclastogenic bone resorption and osteoblastic bone formation in the three types of bone damage remains to be determined.

Here, we show that the JAK inhibitor ameliorated joint erosion, periarticular osteopenia and systemic bone loss in arthritis using comprehensive μCT and histological analyses on CIA mice treated with a JAK inhibitor. Osteoclastogenesis was suppressed in all of the three types of bone damage, while osteoblastic bone formation was promoted only in bone areas distant from the inflamed synovium. All the JAK inhibitors we tested (tofacitinib, baricitinib, upadacitinib, and filgotinib) potently suppressed osteoclastogenesis in the presence of osteoblastic cells. The effect of each JAK inhibitor on osteoclastogenesis in the presence of Th1 cells varied depending on the cytokine milieu, JAK selectivity, and cytokine signaling specificity. These results provide new insights into the potential future use of JAK inhibitors for the treatment of bone damage in RA.

## Methods

### Mice

All animals were maintained under specific pathogen-free conditions. The experiments were approved by the Institutional Review Board at The University of Tokyo.

### Collagen-induced arthritis (CIA)

For CIA, 6- to 8-week-old DBA/1 J male mice (Charles River Laboratories Japan) were used. An emulsion which consisted of 50 μl of chicken type II collagen (Sigma-Aldrich, 4 mg/ml) and 50 μl of adjuvant were injected into the base of the tail at two sites. We added heat-killed Mycobacterium tuberculosis H37Ra (Difco Laboratories, 4 mg/ml) in incomplete Freund’s adjuvant (IFA) (Difco Laboratories). Three weeks after the primary immunization, mice were challenged with the same emulsion as the primary immunization. We judged the development of arthritis in the joint using the following criteria: 0, no joint swelling; 1, swelling of one paw joint; 2, mild swelling of the wrist or ankle; 3, severe swelling of the wrist or ankle. The scores for all of the joints of the forepaws and hind paws as well as wrists and ankles were totaled for each mouse (with a maximum possible score of 12 for each mouse).

### Administration of a JAK inhibitor

CIA mice were administered the JAK inhibitor, upadacitinib (Selleck, ABT-494, 24 mg/kg) in 0.5% methylcellulose, 0.025% Tween 20 solution, or vehicle as a control by oral gavage twice a day from day 7 to day 21 after the 2nd immunization.

### In vitro osteoclast differentiation

Osteoclast precursors were obtained by a culture of primary bone marrow cells purified from 7- to 10-week-old C57BL/6 mice in α-MEM 10% FBS supplemented with 10 ng/ml M-CSF (R&D Systems) for 2 days. Osteoclast precursor cells (2 × 10^4^ cells/well) were then cultured in the presence of JAK inhibitors (tofacitinib: Selleck, CP-690550, baricitinib: Selleck, INCB028050, upadacitinib: Selleck, ABT-494, or filgotinib: Selleck, GLPG0634) for 4 days using a 96-well flat-bottom plate. On day 4, tartrate-resistant acid phosphatase (TRAP) staining was performed. TRAP^+^ multinucleated cells (more than three nuclei) were counted as osteoclasts.

For osteoclastogenesis in the presence of osteoblasts, calvarial cells were isolated from the calvarial bones of newborn mice by enzymatic digestion in α-MEM (Gibco) with 0.1% collagenase (Wako Chemicals) and 0.2% dispase II (Wako Chemicals). Calvarial cells were cultured in α-MEM with 10% FBS for 3 days and used as osteoblastic cells. Osteoclast precursor cells (2 × 10^5^ cells/well) were co-cultured with osteoblastic cells (1 × 10^4^ cells/well) in the presence of 10 nM 1,25-dihydroxy vitamin D3 (1,25D3), 1 μM prostaglandin E2 (PGE2) and the respective JAK inhibitor for 7 days using 24-well flat-bottomed plates. Medium was changed every 3 days. On day 7, TRAP staining was performed and TRAP^+^ multinucleated cells (more than three nuclei) were counted as osteoclasts.

For osteoclastogenesis in the presence of T cells, naïve CD4^+^T cells (CD4^+^CD44^lo^CD62L^hi^) from the spleen and lymph nodes were sorted by FACSAriaIII (BD Biosciences) after enrichment of CD4^+^T cells using anti-CD4 microbeads (Milteny) and LS columns (Milteny Biotech). Naïve CD4^+^T cells were then cultured in the presence of anti-CD3, CD28 microbeads (Dynabeads™ Mouse T-Activator CD3/CD28 for T-Cell Expansion and Activation), IL-12 (10 ng/ml, Peprotech) and anti-IL-4 monoclonal antibody (5 µg/ml, Biolegend) for 3 days, and were used as Th1 cells. Osteoclast precursor cells (1 × 10^5^ cells/well) were co-cultured with Th1 cells (2 × 10^5^ cells/well) in the presence of M-CSF, RANKL (50 ng/ml) and the respective JAK inhibitor for 4 days using a 96-well flat-bottom plate. On day 4, TRAP staining was performed and TRAP^+^ multinucleated cells (more than three nuclei) were counted as osteoclasts.

### Quantitative RT-PCR analysis

Real-time quantitative RT-PCR analysis was performed with a LightCycler (Roche) using SYBR Green (Toyobo). Osteoblastic cells were cultured in the presence of 10 nM 1,25D3, 1 μM PGE2 and the respective JAK inhibitor for 2 days. The level of mRNA expression of osteoblastic cells was normalized by *Gapdh* expression. The following primers were used: *Gapdh*, 5′-TCCACCACCCTGTTGCTGTA-3′ and 5′-ACCACAGTCCATGCCATCAC-3′; *Tnfsf11* 5′-AGCCATTTGCACACCTCAC-3′ and 5′-CGTGGTACCAAGAGGACAGAGT-3′.

### Bone morphometric analysis

Bone morphometric analysis was described previously [26]. Briefly, calcein (Wako) was administered subcutaneously 1 and 5 days before the analysis. The tibia, calcaneus, and vertebrae were fixed in 70% EtOH for 1 week. Toluidine blue and TRAP staining were performed to identify osteoblasts and osteoclasts, respectively.

### Microcomputed tomography analysis

For microcomputed tomography analysis, 3 weeks after the 2nd immunization, the calcaneus, distal femur, and vertebrae of the arthritic mice were subjected to three-dimensional micro-computed tomography. CT scanning was performed using a ScanXmate-A100S Scanner (Comscantechno). Three-dimensional microstructural image data were reconstructed and structural indices were calculated using TRI/3D-BON software (RATOC).

### Statistical analysis

Data were analyzed on GraphPad Prism software version 9.4.1. Statistical tests, *n* values, replicate experiments, and *p* values are all cited in the figures and/or legends. All data are expressed as the mean ± s.e.m. *P* values were calculated using unpaired Student’s *t* test, and one-way ANOVA with the Holm-Sidak multiple comparisons test (*p < 0.05; **p < 0.01; ***p < 0.001; ****p < 0.0001; N.S., not significant, throughout the paper).

## Results

### The JAK inhibitor inhibited joint erosion, periarticular osteopenia, and systemic bone loss under arthritic conditions

**Fig. 1:**
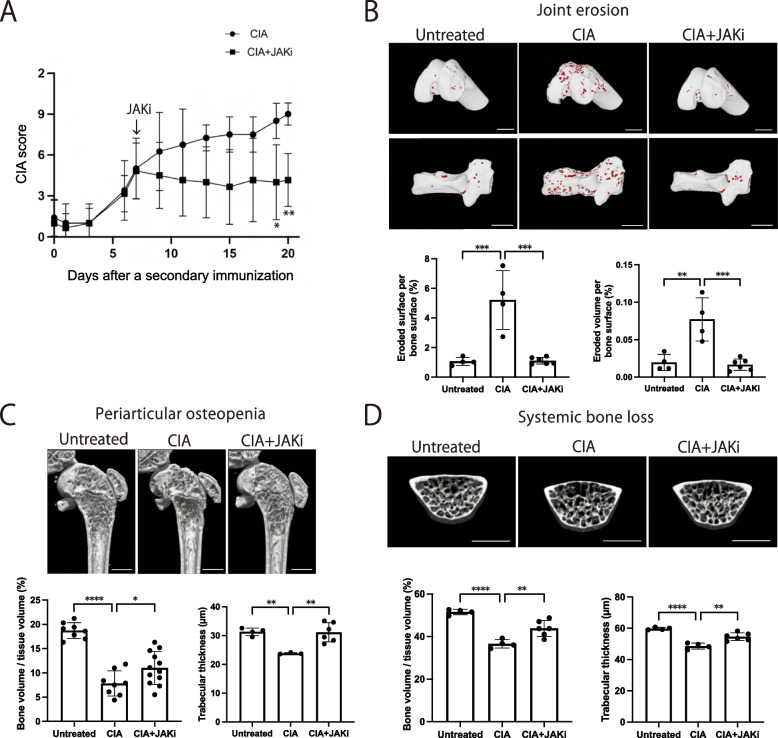
The effect of the JAK inhibitor on bone erosion, periarticular osteopenia and systemic bone loss in autoimmune arthritis. **A** Arthritis score of the CIA mice (*n* = 4) and CIA mice administered the JAK inhibitor upadacitinib (CIA + JAKi mice) (*n* = 6). The JAK inhibitor was administrated 1 week after the secondary immunization. **B** Representative μCT images (upper: knee joint, lower: calcaneocuboid joint), eroded surface (left) and eroded volume (right) per bone surface of the calcaneocuboid joint of untreated (*n* = 4), CIA (*n* = 4) and CIA + JAKi mice (*n* = 6). The red area indicates cavities. **C** Representative μCT images (upper panel), bone volume per tissue volume (BV/TV) (lower left) and trabecular thickness (lower right) of the distal femur of untreated (*n* = 4), CIA (*n* = 4), and CIA + JAKi mice (*n* = 6). **D** Representative μCT images (upper panel), BV/TV (lower left) and trabecular thickness (lower right) of lumbar vertebrae of untreated (*n* = 4), CIA (*n* = 4) and CIA + JAKi mice (*n* = 6). Scale bar: (1 mm) (**B**, **C**, **D**). All data are expressed as the mean ± SEM. *P < 0.05; **P < 0.01; ***P < 0.001; ****p < 0.0001; by unpaired Student’s* t* test (**A**), and one-way ANOVA with the Holm-Sidak multiple comparisons test (**B**–**D**)

We induced collagen-induced arthritis (CIA) in DBA1/J mice and orally administered the JAK inhibitor upadacitinib 1 week after the secondary immunization for 2 weeks to elucidate the effect of the JAK inhibitor on bone damage. The administration of the JAK inhibitor markedly attenuated the CIA severity score based on joint swelling (Fig. 1A). We then evaluated the effect of the JAK inhibitor on three types of bone damage in the CIA mice 3 weeks after the secondary immunization. The erosive surface and volume in the calcaneocuboid and knee joints (analyzed by μCT) were increased in the CIA mice compared to control mice, providing evidence for joint erosion (Fig. 1B, Supplementary Figure S1). The increase in erosive surface and volume was almost abrogated by the JAK inhibitor treatment, indicating effective inhibition (Fig. 1B, Supplementary Figure S1). In addition, μCT analysis showed that bone volume and trabecular thickness in the distal femur were reduced under CIA conditions and that administration of the JAK inhibitor ameliorated this reduction, indicating that the JAK inhibitor suppressed periarticular osteopenia in CIA (Fig. 1C, Supplementary Figure S2). Likewise, the bone volume and trabecular thickness of lumbar vertebrae were reduced in CIA and the reduction was inhibited by the administration of the JAK inhibitor, indicating that the JAK inhibitor also ameliorated systemic bone loss in CIA (Fig. 1D, Supplementary Figure S2).

These results indicate that the JAK inhibitor ameliorated all three types of bone damage in arthritis, while the protective effect against joint erosion was more profound than that on periarticular osteopenia and systemic bone loss.

### The JAK inhibitor reduced the osteoclast number in joint erosion, periarticular osteopenia, and systemic bone loss under arthritic conditions

**Fig. 2:**
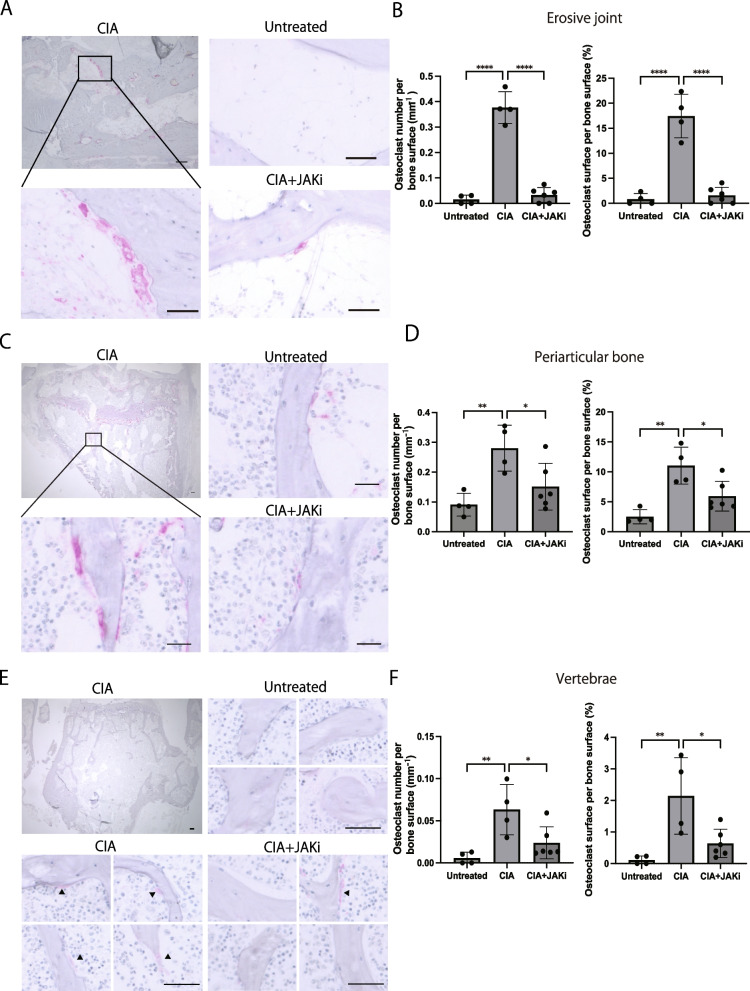
The effect of the JAK inhibitor on osteoclastogenic bone resorption in the three different types of bone damage in autoimmune arthritis. **A**,** B** Representative TRAP^+^ staining (**A**) and the number of TRAP^+^ multinucleated cells per bone surface (**B**) of the calcaneocuboid joint of untreated (*n* = 4), CIA (*n* = 4), and CIA + JAKi mice (*n* = 6). **C**, ** D** Representative TRAP^+^ staining (**C**) and the number of TRAP^+^ multinucleated cells per bone surface (**D**) of periarticular bone (proximal tibia) of untreated (*n* = 4), CIA (*n* = 4) and CIA + JAKi mice (*n* = 6). **E**,** F** Representative TRAP^+^ staining (**E**) and the number of TRAP^+^ multinucleated cells per bone surface (**F**) of lumbar vertebrae of untreated (*n* = 4), CIA (*n* = 4), and CIA + JAKi mice (*n* = 6). Scale bar: (100 μm, upper left; 50 μm, others) (**A**, **E**). (100 μm, upper left; 25 μm, others) (**C**). All data are expressed as the mean ± SEM. *P < 0.05; **P < 0.01; ***P < 0.001; ****p < 0.0001; by one-way ANOVA with the Holm-Sidak multiple comparisons test (**B**, **D**, **F**)

Bone damage in RA results from excessive osteoclastic bone resorption and impaired osteoblastic bone formation [3]. We thus explored whether the suppressive effect of the JAK inhibitor on bone damage is attributable to a decrease in osteoclastic bone resorption and/or upregulation of osteoblastic bone formation. We evaluated the number of TRAP^+^ multinucleated osteoclasts and the osteoclast surface per bone surface in the erosive (calcaneocuboid) joint, periarticular bone (proximal tibia) and lumbar vertebrae of CIA mice treated with the JAK inhibitor. We observed a marked increase in osteoclast number and osteoclast surface per bone surface in the erosive calcaneocuboid joint of CIA mice (Fig. 2A, B). The osteoclast number as well as osteoclast surface per bone surface in the calcaneus was drastically decreased by administration of the JAK inhibitor under CIA conditions (Fig. 2A, B).

The osteoclast number and osteoclast surface per bone surface were also increased in the proximal tibia under CIA conditions, and they were partially reduced by the administration of the JAK inhibitor (Fig. 2C, D). Similarly, in the vertebrae, the osteoclast number and osteoclast surface per bone surface were increased under arthritic condition while they were partially reduced by the JAK inhibitor (Fig. 2E, F).

These results show that the JAK inhibitor inhibited osteoclastogenesis in all three types of bone damage. The increase in osteoclastic bone resorption in CIA mice was almost completely abrogated by the JAK inhibitor in erosive joints whereas it was partially ameliorated in the periarticular bone and vertebrae.

### Osteoblastic bone formation was enhanced in bone areas distal to inflamed synovium

A previous study showed that JAK inhibitors promote osteoblast differentiation and function in vitro [30]. In addition, JAK inhibitors promote bone formation in the joints in certain cases of RA [30]. However, the effect of JAK inhibitors on osteoblastic bone formation in vivo has not yet been quantitatively investigated by bone morphometric analysis.

Since calcein labels newly generated calcified tissue, we injected it twice, with an interval of 4 days, into CIA mice treated with the JAK inhibitor to evaluate osteoblastic bone formation in vivo. The distance between the double-labelled calcein layers indicates the level of bone formation over the 4-day period. The bone formation rate per unit time and per unit bone surface was calculated to evaluate the rate of osteoblastic bone formation. It is reported that bone formation rate was reduced at the navicular bone proximal to inflammatory synovium compared with the navicular bone distant to inflammatory synovium in arthritic joints [34]. In our analysis of the erosive calcaneocuboid joints, we evaluated the effects of the JAK inhibitor on bone formation at the calcaneus adjacent to inflammatory synovium as well as calcaneus distant from inflammatory synovium (Supplementary Figure S3).

**Fig. 3:**
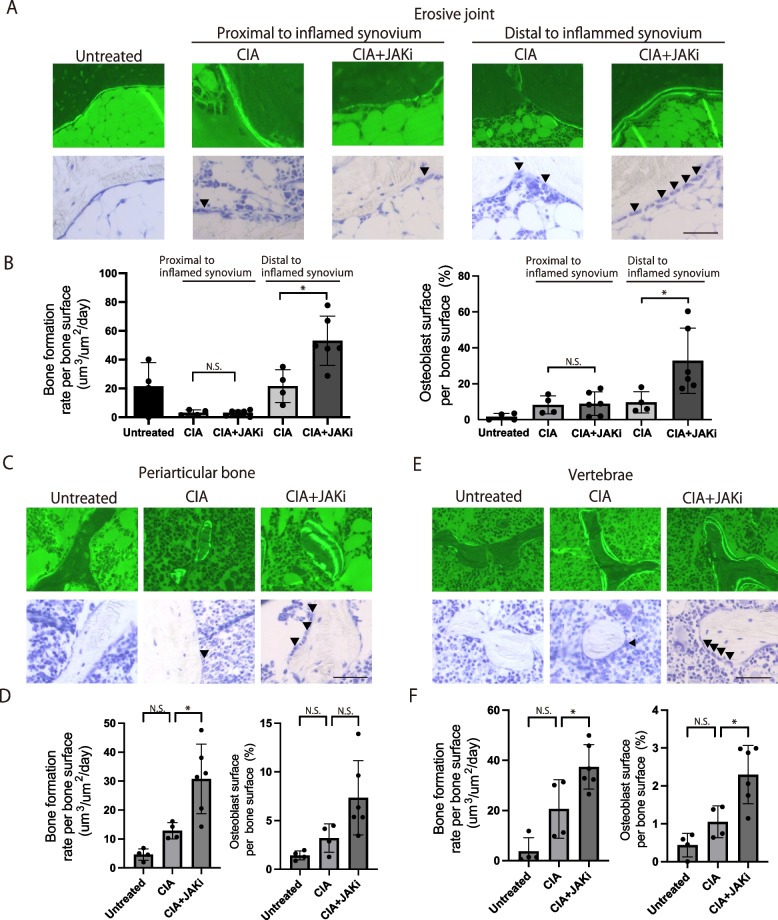
The effect of the JAK inhibitor on osteoblastic bone formation in the three types of bone damage in autoimmune arthritis. **A**, **B** Representative calcein labeling (**A**, upper), toluidine blue staining (**A**, lower), bone formation rate (**B**, left) and osteoblast surface per bone surface of the calcaneus of untreated (*n* = 4), CIA (*n* = 4), and CIA + JAKi mice (*n* = 6). The calcaneus proximal and distal to inflamed synovium were investigated. **C**, **D** Representative calcein labeling (**C**, upper), toluidine blue staining (**C**, lower), bone formation rate (**D**, left), and osteoblast surface per bone surface of the periarticular bone (proximal tibia, **D**, right) of untreated (*n* = 4), CIA (*n* = 4), and CIA + JAKi mice (*n* = 6). **E**,** F** Representative calcein labeling (**E**, upper), toluidine blue staining (**F**, lower), bone formation rate (**F**, left) and osteoblast surface per bone surface of the lumbar vertebrae (**F**, right) of untreated (*n* = 4), CIA (*n* = 4), and CIA + JAKi mice (*n* = 6). Scale bar: (50 μm) (**A**, **C**, **E**). The arrowheads show osteoblasts. All data are expressed as the mean ± SEM. *P < 0.05; by one-way ANOVA with the Holm-Sidak multiple comparisons test (**B**, **D**, **F**) N.S., not significant

There was no difference in the calcaneus adjacent to the inflammatory synovium in terms of the osteoblastic bone formation rate and the number of osteoblasts per bone surface between the JAK inhibitor-treated and vehicle-treated mice under CIA conditions (Fig. 3A, B). Thus, the JAK inhibitor did not influence the bone formation in the inflammatory region. In contrast, in the calcaneus distal to the inflammatory synovium, both the bone formation rate and the number of osteoblasts per bone surface were increased by the JAK inhibitor (Fig. 3A, B).

In the periarticular bone (proximal tibia), osteoblastic bone formation rate per bone surface was increased by the JAK inhibitor under CIA conditions (Fig. 3C, D). In the vertebrae, both the bone formation rate and the osteoblast number per bone surface were increased by the JAK inhibitor under CIA conditions (Fig. 3E, F). These results show that osteoblastic bone formation was enhanced by the JAK inhibitor in the calcaneus distal to the inflammatory synovium in erosive joints, periarticular region of the tibia and vertebrae. This observation is consistent with previous reports that JAK inhibitors promote osteoblast function and mineralization activity in vitro. Importantly, osteoblastic bone formation was not enhanced by the JAK inhibitor at the joint surface where the erosion does occur.

Taken together, the JAK inhibitor markedly reduced bone damage in the erosive joint surface mainly by suppressing osteoclastogenesis, but not promoting osteoblastogenesis. In periarticular bone and vertebrae, the JAK inhibitor partially inhibited bone damage by both suppressing osteoclastic bone resorption and promoting osteoblastic bone formation.

### The effects of various JAK inhibitors on osteoclastogenesis in the RANKL-induced osteoclast formation system and the coculture system of precursor/supporting cells

The effect of the JAK inhibitor we investigated on osteoclastogenesis significantly contributed to its potent bone protective effect, but the effects of various JAK inhibitors on osteoclastogenesis have not been sufficiently examined in multiple osteoclast formation systems. There are several assays for osteoclastogenesis in vitro. Osteoclasts can be differentiated from the monoculture of osteoclast precursor cells by recombinant RANKL stimulation. This RANKL-induced osteoclast formation system is suitable for investigating the direct effect of JAK inhibitors on osteoclast precursor cells. The co-culture of osteoclast precursor cells with osteoclastogenesis-supporting mesenchymal cells, such as osteoblastic cells, enables an investigation of the effect of JAK inhibitors on osteoclastogenesis-supporting cells as well as precursor cells.

**Fig. 4:**
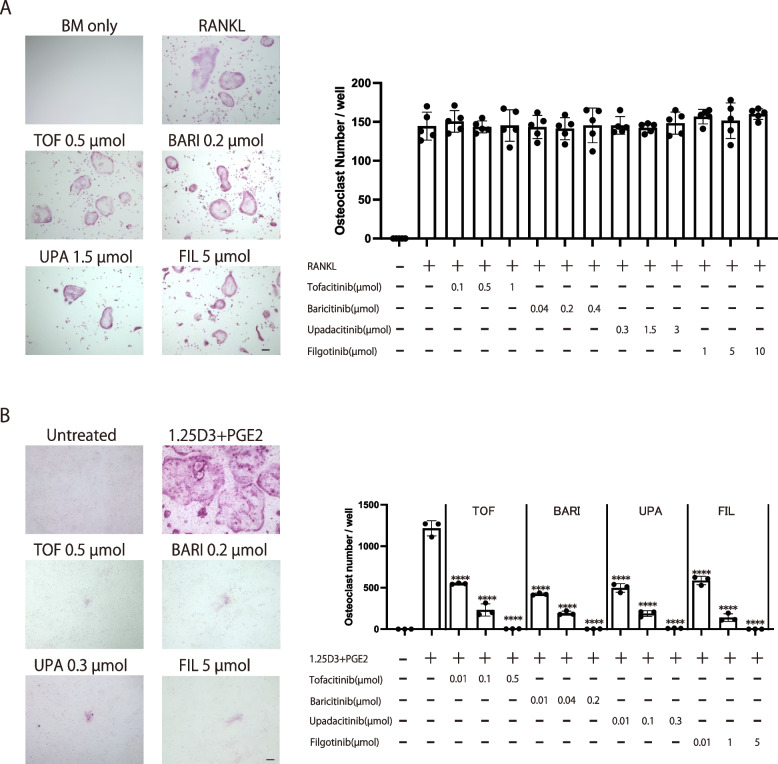
The effects of the various JAK inhibitors on osteoclastogenesis in a monoculture of osteoclast precursor cells and coculture system of precursors with supporting cells. **A** Representative TRAP staining (left) and number of TRAP^+^ multinucleated cells (right) in the presence of the respective JAK inhibitor in the monoculture of osteoclast precursors. **B** Representative TRAP staining (left) and number of TRAP^+^ multinucleated cells (right) in the presence of the respective JAK inhibitor in the coculture of osteoclast precursors and osteoblastic cells. Scale bar: (100 μm). All data are expressed as the mean ± SEM. ****p < 0.0001; by one-way ANOVA with the Holm-Sidak multiple comparisons test

The currently approved JAK inhibitors include tofacitinib, baricitinib, upadacitinib, and filgotinib. We added each JAK inhibitor (tofacitinib, baricitinib, upadacitinib, and filgotinib) to the RANKL-induced osteoclast formation system separately and evaluated the number of TRAP^+^ multinucleated osteoclasts. The concentration of each JAK inhibitor was adjusted according to the previous studies based on the daily dosage of JAK inhibitors used for RA treatment [30, 31, 35]. RANKL induced an efficient formation of TRAP^+^ multinucleated cells (Fig. 4A). The number of TRAP^+^ multinucleated cells was unchanged in the presence of tofacitinib, baricitinib, upadacitinib, or filgotinib, indicating that none of these JAK inhibitors affected osteoclastogenesis in this RANKL-induced osteoclast formation system (Fig. 4A). In contrast, when each JAK inhibitor was respectively added to the co-culture system of osteoclast precursor cells with osteoblastic cells, the number of TRAP^+^ multinucleated cells was decreased in a dose-dependent manner (Fig. 4B). All of the JAK inhibitors suppressed osteoclastogenesis in the presence of osteoblastic cells to a similar extent (Fig. 4B). RANKL and M-CSF produced by osteoblastic cells are essential for osteoclastogenesis in this co-culture system. It is reported that a JAK inhibitor baricitinib inhibits osteoclastogenesis by inhibiting RANKL, but not M-CSF expression in osteoblastic cells [31]. In line with this, RANKL expression in osteoblastic cells was inhibited in the presence of various JAK inhibitors in a dose dependent manner (Supplementary Figure S4). Taken together, it is suggested that all of these JAK inhibitors are capable of suppressing osteoclastogenesis, possibly by inhibiting RANKL expression on mesenchymal osteoclastogenesis-supporting cells.

### The effects of various JAK inhibitors on osteoclastogenesis in the RANKL-induced osteoclast formation system cocultured with Th1 cells

When T cells are added into the RANKL-induced osteoclast formation system, it is possible to estimate the effect of JAK inhibitors on osteoclast precursor cells under certain conditions in which T cells are abundantly present, such as inflammatory synovium [6, 7, 36, 37].

While all of the JAK inhibitors show similar effect on JAK1 inhibition and have similar efficacy in RA, the incidence of adverse events varies, which may be due to the different selectivity for other JAKs or cytokine signaling specificity [18, 38, 39]. A recent study using immune cells from the peripheral blood of RA patients reported that the selective JAK1 inhibitor filgotinib inhibited the IFN-α and IL-6 signaling pathways to a similar extent as other JAK inhibitors, while it inhibited IFN-γ signaling and JAK2-mediated pathways to a lesser extent [35]. Among the proinflammatory cytokines present in RA synovium, such as IL-6, TNF, IL-17, and IFN-γ, IFN-γ potently inhibits osteoclast differentiation [37]. We have been studied the inhibitory effect of IFN-γ on osteoclastogenesis and reported that Th1 cells, one of the major cellular sources of IFN-γ in RA synovium, inhibit osteoclastogenesis in an IFN-γ-dependent manner [37].

**Fig. 5:**
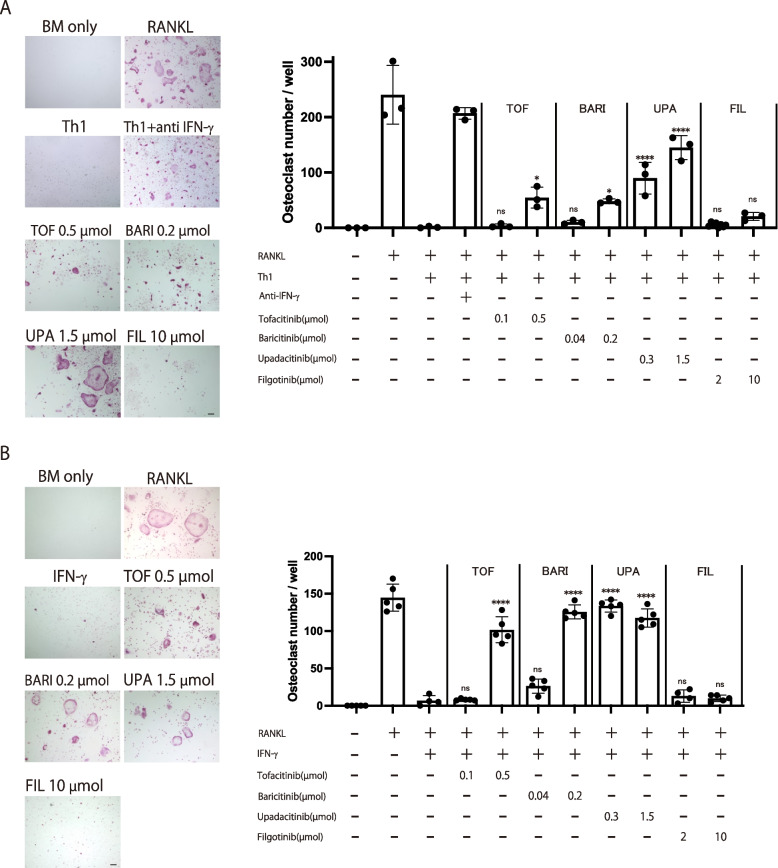
The effects of the various JAK inhibitors on osteoclastogenesis in a RANKL-induced osteoclast formation system cocultured with Th1 cells. **A** Representative TRAP staining (left) and number of TRAP^+^ multinucleated cells (right) in the presence of the respective JAK inhibitor in the RANKL-induced osteoclast formation system cocultured with Th1 cells. **B** Representative TRAP staining (left) and number of TRAP^+^ multinucleated cells (right) in the presence of the respective JAK inhibitor and IFN-γ in the RANKL-induced osteoclast formation system. Scale bar: (100 µm). All data are expressed as the mean ± SEM. *P < 0.05, ****p < 0.0001; by one-way ANOVA with the Holm-Sidak’s multiple comparisons test

To investigate the effect of JAK inhibitors on osteoclastogenesis under Th1 cell-abundant conditions, we added each JAK inhibitor into the co-culture system of osteoclast precursors with Th1 cells. In the absence of JAK inhibitors, we observed that RANKL induced efficient TRAP^+^ multinucleated cell formation, an effect which was suppressed by the addition of Th1 cells. Consistent with a previous report, the addition of an anti-IFN-γ antibody abrogated the suppressive effect, confirming that Th1 cells inhibit osteoclastogenesis in an IFN-γ-dependent manner (Fig. 5A) [37]. Th1-mediated suppression of osteoclastogenesis was abrogated by tofacitinib, baricitinib or upadacitinib in a dose-dependent manner, possibly because these JAK inhibitors suppress IFN-γ signaling in osteoclast precursor cells. In contrast, osteoclast formation remained impaired in the presence of filgotinib, probably due to a lesser inhibitory effect on IFN-γ signaling compared with the other JAK inhibitors (Fig. 5A).

In an effort to conclusively demonstrate that the effect of JAK inhibitors depends on IFN-γ signaling specificity, we replaced Th1 cells with IFN-γ. IFN-γ (5 ng/ml) completely inhibited the formation of TRAP^+^ multinucleated osteoclasts. This IFN-γ-mediated suppression of osteoclastogenesis was abrogated by tofacitinib, baricitinib or upadacitinib in a dose-dependent manner. In contrast, osteoclastogenesis remained suppressed in the presence of filgotinib even at a high concentration, confirming that the effect of JAK inhibitors on the Th1-mediated suppression of osteoclast formation relies on its ability to inhibit IFN-γ signaling (Fig. 5B).

Taken together, all the JAK inhibitors suppressed osteoclastogenesis in the presence of osteoblastic cells to a similar extent in vitro. Suppression of osteoclastogenesis by Th1 cells was abrogated by tofacitinib, baricitinib, and upadacitinib, while filgotinib did not affect the suppression, possibly because it inhibits IFN-γ signaling to a lesser extent than the other JAK inhibitors.

## Discussion

To the best of our knowledge this is the first study that elucidates the comprehensive effect of JAK inhibitors on bone loss, osteoclastic bone resorption and osteoblastic bone formation in the three forms of bone damage in autoimmune arthritis: joint erosion, periarticular osteopenia and systemic bone loss. While the effects of JAK inhibitors on immune cells and synovial fibroblasts have been extensively studied, their effect on bone cells has been reported in only a few studies [18–25]. In particular, the details of the effect of JAK inhibitors on osteoclastogenic bone resorption and osteoblastic bone formation in all the types of bone damage in vivo had needed clarification.

We report here that the JAK inhibitor inhibited all three types of bone damage: joint erosion, periarticular osteopenia and systemic bone loss damage in an autoimmune arthritis animal model using the authentic bone morphometric analyses and µCT. The JAK inhibitor suppressed osteoclastogenesis in all three types of bone damage, while the effect on erosive joints was more potent than that on periarticular bone or vertebrae. The JAK inhibitor failed to promote bone formation at sites proximal to inflamed synovium in the erosive joints, while it promoted osteoblastic bone formation at sites distal to inflamed synovium in the erosive joints, periarticular bone, and vertebrae. Although the reasons why upadacitinib could not promote bone formation at sites proximal to the inflamed synovium remain unclear, it is suggested that inhibitory factors for osteoblastogenesis such as TNF and DKK-1, may be abundant at sites proximal to inflamed synovium and inhibit osteoblastic bone formation irrespectively of JAK/STAT signaling pathways [40].

These results indicate that the JAK inhibitor suppressed joint erosion mainly by inhibiting osteoclastogenesis, while ameliorating periarticular osteopenia and systemic bone loss by inhibiting osteoclastogenesis and promoting osteoblastogenesis. Thus, it is suggested that the effect of the JAK inhibitor on osteoclastogenesis and osteoblastogenesis under arthritic conditions depends on the type and area of the bones affected. The JAK inhibitor was administered for 2 weeks in this study. It will be important to evaluate the effect of the JAK inhibitors administered for longer periods in future studies. Since the longevity of osteoblasts is longer than osteoclast, it is possible that number of osteoblasts may be increased and thus osteoblastic bone formation may be upregulated especially in the calcaneus distal to inflammatory synovium in the calcaneocuboid joints, periarticular region of the tibia and vertebrae if the JAK inhibitors were administered for longer periods.

The studies we performed showed that all of the JAK inhibitors suppressed osteoclastogenesis in the presence of osteoblastic cells in vitro. In light of the fact that JAK inhibitors suppress osteoclastogenesis by inhibiting RANKL expression of osteoblastic cells, it is suggested that the inhibitory effect in vivo may be attributed to the inhibition of RANKL expression of osteoclast-supporting mesenchymal cells such as osteoblasts and synovial fibroblasts [31]. A JAK inhibitor was recently reported to inhibit osteoclastogenesis by suppressing both the migration of osteoclast precursor cells and the function of osteoclasts in vivo at the site of LPS injection [32]. In the future, it will be important to evaluate the extent to which each factor contributes to the suppressive effect of JAK inhibitors on osteoclastogenesis under arthritic conditions in vivo.

The effect of the JAK inhibitor on osteoclastogenesis was stronger at erosive joints compared to other bone regions. It may be partly because periarticular osteopenia and systemic bone loss may precede joint erosion when we start administration of the JAK inhibitor in this study [12]. It is suggested joint erosion may be induced mainly by inflammation in JAK/STAT signaling pathway-dependent way, whereas periarticular osteopenia and systemic bone loss can be induced by autoimmunity via immune complexes in a JAK/STAT signaling pathway-independent ways. Especially in erosive joints, it is suggested that RANKL expression in synovial fibroblasts, the migration of osteoclast precursor cells and the function of osteoclasts may be dependent on JAK/STAT signaling pathways.

While the approved JAK inhibitors exert similar effects on RA, the adverse events vary, most likely due to the different selectivity for JAKs and specificity of the cytokine signaling pathways [35]. In this study, we found that the inhibition of osteoclastogenesis by Th1 cells was abrogated by most of the JAK inhibitors, with only filgotinib not reversing this inhibition, possibly because it inhibits IFN-γ signaling to a lesser extent than the other JAK inhibitors. While both filgotinib and upadacitinib have high JAK1 selectivity to the similar extent, filgotinib did not affect the suppression of osteoclastogenesis by Th1 cells, possibly because it impairs an IFN-γ signaling pathway to a lesser extent compared with upadacitinib. Thus, these different effects on osteoclastogenesis may be due to differences in the specificity of the cytokine signaling pathways rather than the JAK selectivity. Thus, it is suggested the effect of JAK inhibitors on osteoclastogenesis under inflammatory conditions may vary depending on the cytokine milieu, JAK selectivity and specificity of the cytokine signaling pathways.

Since IFN-γ signaling is also important for host defense against pathogens such as herpes virus, it will be important to choose which JAK inhibitor is to be used for RA treatment based on the cytokine milieu in the synovium and the risk of viral infection in RA patients. Considering that filgotinib inhibits IFN-γ signaling pathway to a lesser extent compared with other JAK inhibitors including upadacitinib, it is suggested that filgotinib may effectively inhibit joint erosion in RA patients with IFN-γ-abundant synovium and that it may be appropriate for RA patients with a high risk of viral infection. Although this study provides the opportunity to consider better therapeutic strategies based on the cytokine signaling specificity of JAK inhibitors, further evaluation is necessary for determining the optimal usage of JAK inhibitors. The findings presented in this study overall provide new insights into the future use of JAK inhibitors for the treatment of bone damage in RA.

## Conclusion

JAK inhibitors effectively inhibit inflammation and joint erosion in RA. However, their effects on osteoclastic bone resorption and osteoblastic bone formation in all the types of bone damage in autoimmune arthritis have not been previously evaluated in depth. This study shows that the JAK inhibitor suppressed joint erosion mainly by inhibiting osteoclastogenesis, while it ameliorated periarticular osteopenia and systemic bone loss by both inhibiting osteoclastogenesis and promoting osteoblastogenesis. While the currently available JAK inhibitors are capable of inhibiting osteoclastogenesis, the suppressive effects under inflammatory environments may vary depending on the cytokine milieu, JAK selectivity and the specificity of the cytokine signaling pathways. The findings reported here should contribute to the strategic therapeutic use of JAK inhibitors against bone damage in RA.

### Supplementary Information

**Additional file 1: Supplementary Figure S1.** The effect of the JAK inhibitor on bone erosion in the knee joint. **Supplementary Figure S2.** The effect of the JAK inhibitor on periarticular osteopenia and systemic bone loss. **Supplementary Figure S3.** Bone areas of the calcaneus proximal and distal to the inflammatory synovium assessed for the effect of the JAK inhibitor on bone formation. **Supplementary Figure S4.** RANKL expression by osteoblastic cells in the presence of various JAK inhibitors in the co-culture system.

## Data Availability

All the data that support the plots within this paper are available in the main text.

## Abbreviations

RA: Rheumatoid arthritis
CIA: Collagen-induced arthritis
μCT: Micro-computed tomography
JAK: Janus kinase
STAT: Signal transducer and activator of transcription
RANKL: Receptor activator of NF-κB ligand
M-CSF: Macrophage colony-stimulating factor
bDMARD: Biologic disease modifying anti rheumatic drugs
tsDMARD: Targeted synthetic disease modifying anti rheumatic drugs
TNF: Tumor necrosis factor
IL: Interleukin
IFN: Interferon
DKK-1: Dickkopf-1
IFA: Incomplete Freund’s adjuvant
TRAP: Tartrate-resistant acid phosphatase
1,25D3: 1,25-Dihydroxy vitamin D3
PGE2: Prostaglandin E2
