# Supplementary material for: Effect of JAK inhibitors on the three forms of bone damage in autoimmune arthritis: joint erosion, periarticular osteopenia, and systemic bone loss
Source: Inflamm Regen. 2023 Sep 19;43:44. doi: 10.1186/s41232-023-00293-3 (PMC10507845; doi:10.1186/s41232-023-00293-3)
Supplement: Supplementary file 1 — Additional file 1: Supplementary Figure S1. The effect of the JAK inhibitor on bone erosion in the knee joint. Supplementary Figure S2. The effect of the JAK inhibitor on periarticular osteopenia and systemic bone loss. Supplementary Figure S3. Bone areas of the calcaneus proximal and distal to the inflammatory synovium assessed for the effect of the JAK inhibitor on bone formation. Supplementary Figure S4. RANKL expression by osteoblastic cells in the presence of various JAK inhibitors in the co-culture system. [file 41232_2023_293_MOESM1_ESM.pdf]

Supplementary Figure 1

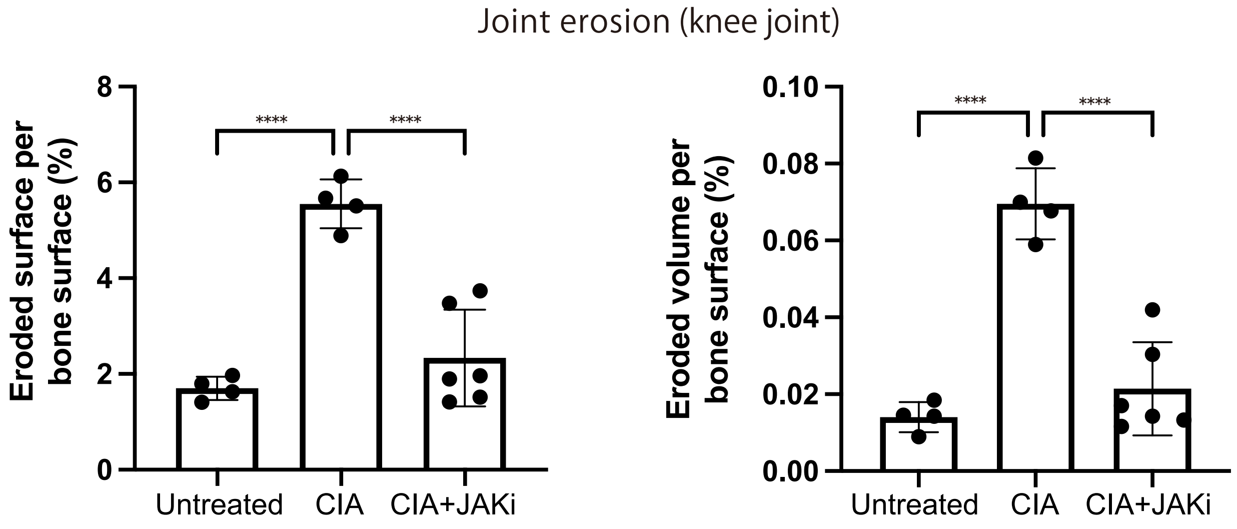

Supplementary Figure 1 The effect of the JAK inhibitor on bone erosion in the knee joint

Eroded surface (left) and volume (right) per bone surface of the knee joint of untreated (n=4), CIA (n=4) and CIA+JAKi mice (n=6). All data are expressed as the mean ± SEM. ****$p < 0.0001$; by one-way ANOVA with the Holm-Sidak multiple comparisons test.

Supplementary Figure 2

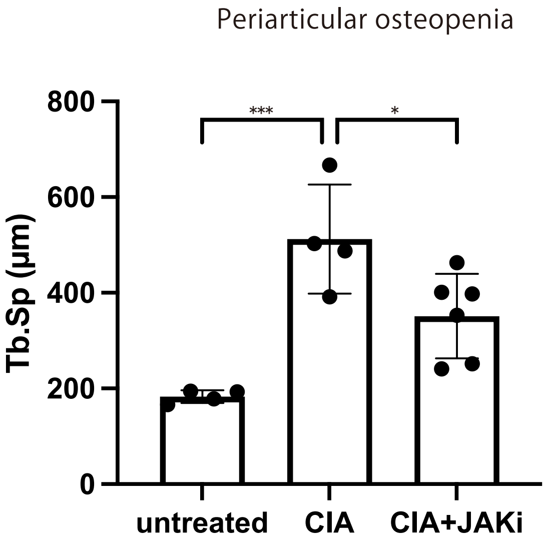

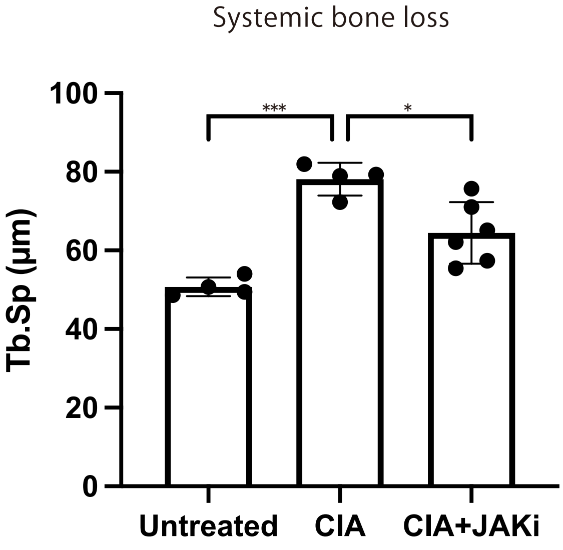

Supplementary Figure 2 The effect of the JAK inhibitor on periarticular osteopenia and systemic bone loss

Trabecular separation (Tb. Sp) of the periarticular bone (distal femur, left) and the lumbar vertebrae (right) of untreated (n=4), CIA (n=4) and CIA+JAKi mice (n=6). All data are expressed as the mean ± SEM. ****$p < 0.0001$; by one-way ANOVA with the Holm - Sidak multiple comparisons test.

Supplementary Figure 3

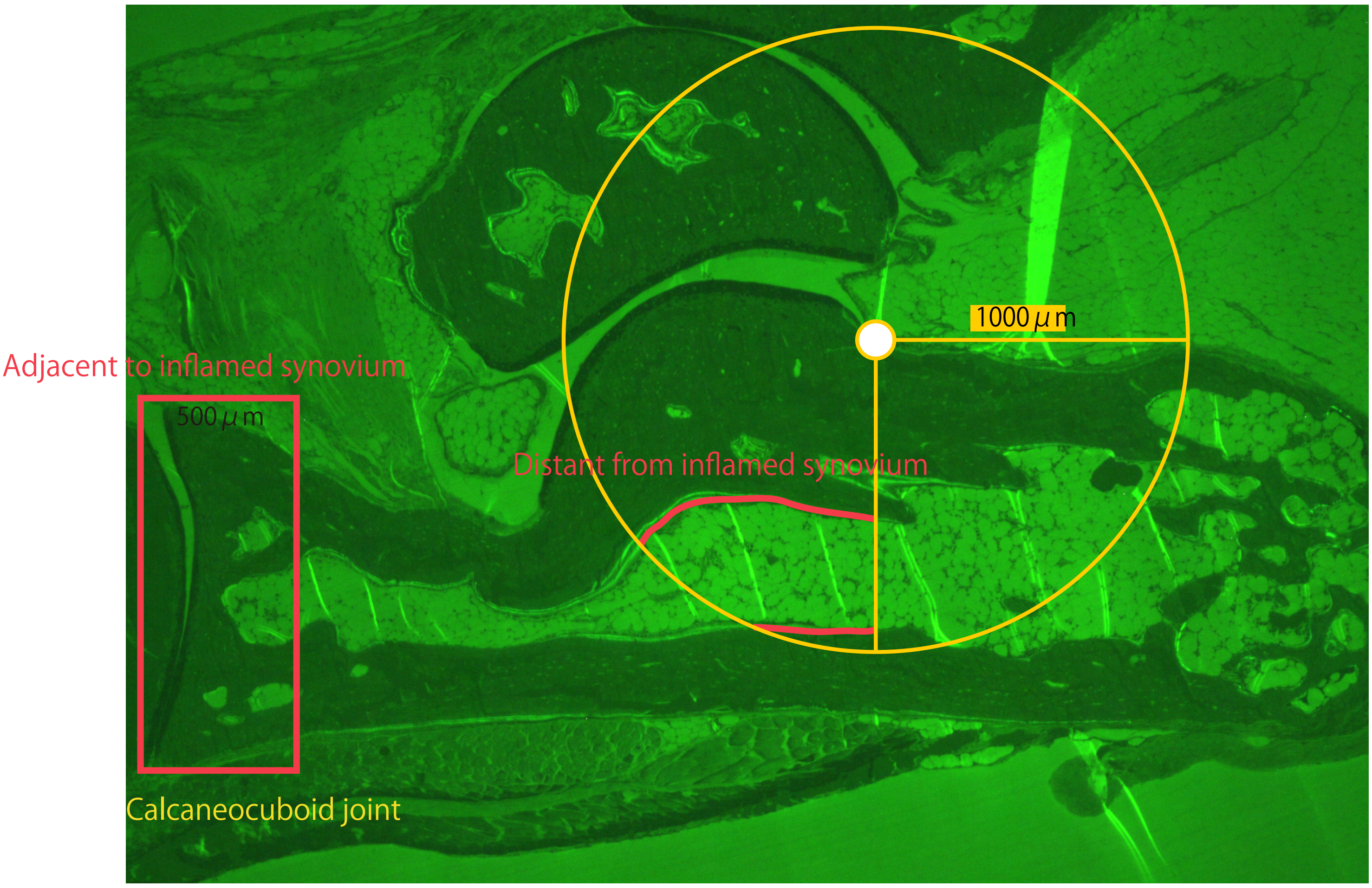

Supplementary Figure 3 Bone areas of the calcaneus proximal and distal to the inflammatory synovium assessed for the effect of the JAK inhibitor on bone formation

The assessed bone areas of calcaneus proximal (red square) and distal to inflammatory synovium (red line) are shown. The calcaneus proximal to inflammatory synovium was defined as the bone area within 500 µm of the distal edge of the calcaneus in the erosive calcaneocuboid joints (red square). The calcaneus distal to inflammatory synovium was defined as the bone area proximal to the bone marrow (red line) within the left half of a 1000 µm circle with its center at the posterior end of the subtalar joint (yellow).

Supplementary Figure 4

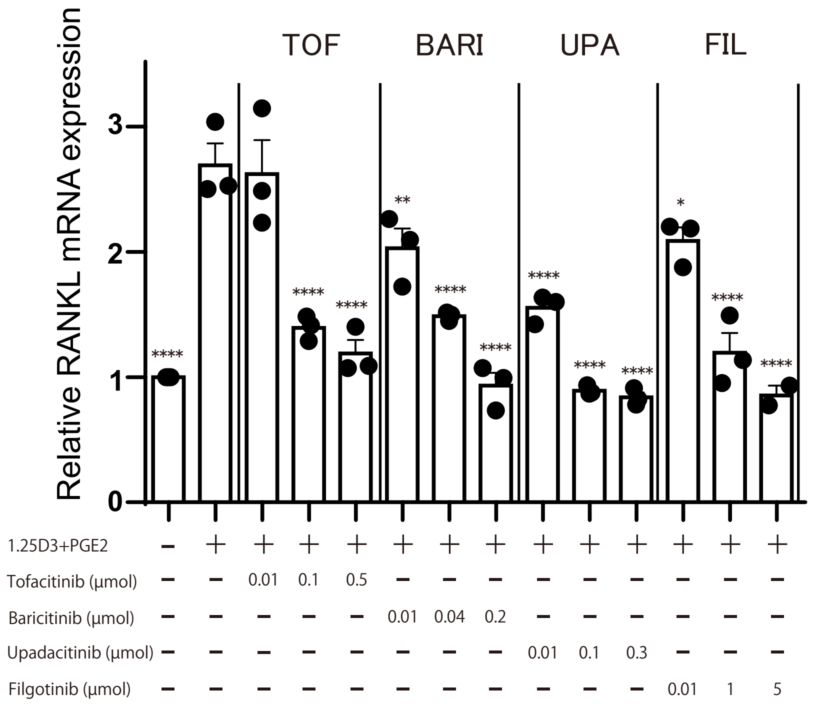

Supplementary Figure 4: RANKL expression by osteoblastic cells in the presence of various JAK inhibitors in the co-culture system.
Osteoblastic cells were cultured in the presence of 1,25D3, PGE2 and the respective JAK inhibitor for 2 days. RANKL mRNA expression of osteoblastic cells was evaluated by qRT-PCR.
All data are expressed as the mean ± SEM. *$p < 0.05$,**$p < 0.01$,****$p < 0.0001$; by one-way ANOVA with the Holm-Sidak multiple comparisons test.
